# Yoga for Depressive Disorder: A Systematic Review and Meta-Analysis

**DOI:** 10.1155/da/6071055

**Published:** 2024-12-19

**Authors:** Alina Moosburner, Holger Cramer, Mirela Bilc, Johanna Triana, Dennis Anheyer

**Affiliations:** ^1^Institute of General Practice and Interprofessional Care, University Hospital Tübingen, Tübingen, Germany; ^2^Robert Bosch Center for Integrative Medicine and Health, Stuttgart, Germany; ^3^Department of Natural and Integrative Medicine, Robert-Bosch-Hospital, Stuttgart, Germany; ^4^Department of Psychology and Psychotherapy, University Witten/Herdecke, Witten, Germany

## Abstract

**Background:** The prevalence of depression has been increasing sharply. Given the existing treatment gap and the high prevalence of nonresponders to conventional therapies, the potential of complementary medicine becomes clear. The effect of yoga on depression has already been studied, but its efficacy in manifest depressive disorders remains unclear.

**Objective:** To update and evaluate the current state of evidence for yoga as a therapy option for depressive disorders.

**Methods:** PubMed/Medline, Cochrane Library, Scopus, PsycINFO, and BASE were searched systematically. Randomized controlled trials (RCTs), including participants with depressive disorders, were eligible. Analyses were conducted for active and passive control groups separately and for subgroups of major depressive disorder (MDD) and mixed samples. The risk of bias was assessed using the Cochrane risk of bias tool 2.0. Primary outcomes were the severity of depression and remission rates, and secondary outcomes were health-related quality of life and adverse events. The quality of evidence was assessed according to Grading of Recommendations, Assessment, Development, and Evaluations (GRADE).

**Results:** Twenty-four studies (*n* = 1395) were included; of those, 20 studies (*n* = 1333) were meta-analyzed. Yoga showed a statistically significant short-term effect on depression severity when compared to passive control (standardized mean difference [SMD] = −0.43, 95% confidence interval [CI] = [−0.80; −0.07]) but not when compared to active control (SMD = −0.22, 95% CI  = [−0.67; 0.23]). Regarding remission rates, statistically significant effects were observed when comparing yoga to passive (odds ratio [OR] = 3.20; 95% CI = [1.45; 7.10]) as well as to active control (OR = 2.04; 95% CI = [1.13; 3.69]). No differences on safety outcomes were observed for passive (OR = 1.00, 95% CI = [0.10; 9.98]) as well as for active control (OR  = 0.80, 95% CI = [0.08; 8.09]). The quality of evidence ranged from moderate to very low. Due to the heterogeneity of outcome reporting, no meta-analysis for quality of life was possible.

**Conclusion:** Yoga is an effective therapy approach for reducing depression severity when compared to passive control and obtains higher remission rates when compared to active and passive controls. Quality of evidence is inconsistent, but given the positive risk–benefit ratio of the intervention and the urge for therapy options for depression, yoga should be considered as a possible treatment option, particularly for MDD patients.

## 1. Introduction

Depressive disorders are highly prevalent and burdensome mental health disorders, affecting about 3.4% of the population worldwide. The prevalence has further increased following the COVID-19 pandemic [1–4]. One of the most commonly diagnosed forms is major depressive disorder (MDD), characterized by at least 2 weeks of depressed mood and/or loss of interest or pleasure, often accompanied by weight and psychomotor changes, sleep disturbances, fatigue, decreased concentration or thoughts of death [5].

Standard treatments for depression include psychotherapy, pharmacotherapy (e.g., selective serotonin reuptake inhibitors), or their combination. Among MDD patients, a prevalence of 20% to 40% of nonresponders has to be considered [6]. In combination with a large treatment gap, the urge of an effective, widespread, and easily accessible therapy becomes clear [7, 8].

One possible approach is complementary medicine [9, 10]. It comprises various psychological and physiological therapeutic approaches and aims to complement conventional medicine to build an integrative therapy. One such approach is yoga. Its practice is increasing, often aiming to improve wellbeing and mental health. It represents a form of physical activity that is often considered more gentle and accessible than other forms of exercise, especially for people suffering from depression [11, 12]. There is a high prevalence of yoga practice among patients affected by depression [13]. In the Western world, yoga is mostly associated with physical postures (asanas), breathing techniques (pranayama), and meditation (dhyana) [14]. The positive effects of yoga on multiple health issues or their symptoms have already been described [15–23].

Also, the effect of yoga interventions on depression has already been investigated in prior systematic research. Reviews exhibit the potential benefits of yoga for reducing depressive symptoms [24, 25], and in meta-analyses, yoga interventions show significant beneficial effects [26, 27]. The positive effect of yoga may extend beyond the effect of physical activity alone. Meditation, breathing exercises [28], and relaxation [29] seem to contribute to the antidepressive effect. Various potential mechanisms of action have been described [30, 31].

We conducted the current systematic review and meta-analysis to evaluate the evidence for the effect of yoga on depression severity, remission rates, quality of life, and its safety in people with a clinically diagnosed depressive disorder.

## 2. Methods

The review was designed and conducted in accordance with the Preferred Reporting Items for Systematic Reviews and Meta-Analyses (PRISMA) guidelines [32, 33] (Supporting Information 5) and the recommendations of the Cochrane Collaboration [34]. The study was registered prospectively on INPLASY (protocol-number: 202330033) [35].

### 2.1. Eligibility Criteria

#### 2.1.1. Study Types

Randomized controlled trials (RCTs) published in English, German, Italian, Spanish, French, Croatian, Russian, Bosnian, and Serbian were included.

#### 2.1.2. Participants

Studies on participants with a clinical diagnosis of a depressive disorder (MDD or other) according to DSM-IV/V or ICD-10/11 and a minimum age of 18 years were included. No restrictions regarding gender and ethnicity were made. Studies investigating depression as a comorbidity of a different primary disorder were excluded. Studies including pregnant participants were eligible if the diagnosis of depression was unrelated to pregnancy.

#### 2.1.3. Interventions

Any type of yoga intervention was eligible. Related interventions, such as mindfulness-based therapies, were included if they mainly consisted of yoga. When eligibility was unclear, authors were contacted. No restrictions regarding control interventions were defined.

#### 2.1.4. Outcome Measures

Primary outcomes were depression severity, as assessed with validated questionnaires, and remission rates. If more than one severity score was reported, the one most commonly used was included. Secondary outcomes were health-related quality of life, assessed with validated questionnaires, and safety of the intervention, assessed with reporting of adverse and/or serious adverse events. Short-, intermediate-, and long-term outcomes were defined as closest to 12, 24, and more than 24 weeks after randomization.

### 2.2. Search Strategy

The databases Medline, Cochrane, Scopus, PsycINFO, and BASE (gray literature) were searched. The search strategy was built around the keywords yoga and depression (Supporting Information 1). Titles, abstracts, and full texts were screened by two authors independently (A.M. and J.T.). Disagreements were discussed with a third author (D.A.) until a consensus was reached.

### 2.3. Data Extraction Management

The studies' characteristics and results were extracted by two authors independently (A.M. and M.B.). Discrepancies were discussed with a third author (D.A.) until a consensus was reached.

### 2.4. Risk of Bias in Individual Studies

To assess individual risk of bias, the Cochrane Risk of Bias Tool 2.0 [36] was used by two authors independently (A.M. and D.A.). Five subdomains are judged as low, some concerns, and high. Discrepancies were discussed with a third author (H.C.) until a consensus was reached.

### 2.5. Overall Effect Sizes

When a minimum of two studies were accessible for an outcome, combined analyses were executed using R software version 4.2.3 (R Foundation for Statistical Computing, Vienna, Austria, URL: https://cran.r-project.org) along with the “meta” [37]. For continuous outcomes, standardized mean differences (SMDs) accompanied by 95% confidence intervals (CIs) were computed [34]. For dichotomous outcomes, odds ratios (ORs) with 95% CIs were computed. In instances of missing data, authors were contacted [34].

Random-effects models were determined using the inverse variance approach for continuous outcomes and the Mantel–Haenszel method for dichotomous outcomes [34]. Additionally, the Hartung–Knapp small-sample correction was implemented. It offers a more appropriate handling of uncertainty when consolidating treatment effects from a limited number of heterogeneous studies [38–41]. The Cohen categories were applied to gauge the magnitude of the overall effect size [42]. For depression severity, the clinical relevance of change and minimum clinically important differences (MCID) were calculated based on a relative clinically relevant change of 23% [43] with mean values of a norm population [44]. Common language effect sizes (CLES) were calculated for all outcomes [45].

### 2.6. Subgroups, Sensitivity Analyses, and Meta-Regression

Analyses were conducted for active and passive control groups separately. Subgroups were defined based on diagnosis (MDD vs. mixed samples) and for studies including women only. Sensitivity analyses were carried out considering studies with low risk of bias.

### 2.7. Heterogeneity

The assessment of statistical heterogeneity among studies was undertaken using the *I*^2^ and *t*^2^ statistics. *I*^2^ quantifies the proportion of heterogeneity in treatment evaluations, whereas *t*^2^ elucidates the fundamental heterogeneity among studies and does not inherently escalate with the augmentation of study count or sample size. *I*^2^ was interpreted as 0%–24% low, 25%–49% moderate, 50%–74% substantial, and 75%–100% considerable heterogeneity [46]. For *t*^2^ statistics, the restricted maximum-likelihood estimator was applied [34].

### 2.8. Quality of Evidence

To assess the quality of the evidence, outcomes of overall effects were evaluated by Grading of Recommendations, Assessment, Development, and Evaluations (GRADE) [47]. Five domains result in an overall rating of certainty (very low, low, moderate, and high). Assessment was done by two authors independently (A.M. and D.A.). Discrepancies were discussed with a third author (H.C.) until a consensus was reached.

### 2.9. Risk of Bias Across Studies

If a minimum of 10 studies were included in a meta-analysis, publication bias was assessed through visual examination of funnel plots. A linear regression test (Egger‘s test) was conducted to evaluate publication bias.

## 3. Results

### 3.1. Literature Search

**Figure 1:**
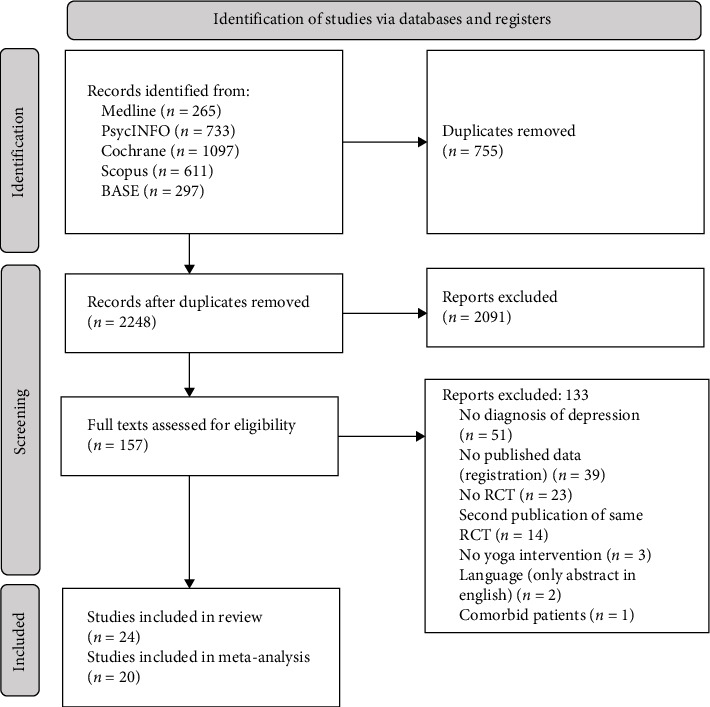
Flowchart of study inclusion according to PRISMA. PRISMA, Preferred Reporting Items for Systematic Reviews and Meta-Analyses; RCT, randomized controlled trial.

Literature search retrieved 2248 records after the removal of duplicates. Twenty-four articles reporting 22 RCTs with a total of 1395 participants were included in the systematic review, and 20 RCTs with 1333 participants were included in the meta-analysis (Figure 1). In case of the same RCT being published twice, the one with the bigger sample [48, 49], or reporting the more common outcome data [50, 51] was included. Two studies were included in the qualitative analysis but not in the meta-analysis.

### 3.2. Study Characteristics

Characteristics of the included studies are shown in Table 1.

#### 3.2.1. Setting and Participants

Most studies originated from North America [48–50, 57, 58, 61, 64, 66, 69, 70] and Asia [52, 55, 59, 62, 65, 67, 68, 72], followed by Europe [54, 56, 63, 71].

In 16 studies, patients with a diagnosis of MDD [49, 50, 52, 54, 56, 59, 62–68, 70–72] were included, six included mixed population [48, 55, 57, 58, 61, 69].

Average age ranged from 24.9 to 51.0 years (median: 36.7 years). The proportion of female participants was 23.8%–100% (median: 76.8%), with six studies including women only [48, 58, 61, 64, 69, 71]. Mostly white participants were included [48–50, 54, 57, 61, 66, 69, 70].

#### 3.2.2. Intervention

The yoga-type most commonly used was Hatha Yoga [48, 49, 57, 59, 63, 66, 68, 70, 72]. Some studies used mindfulness-based yoga concepts [56, 64, 71], yoga interventions specifically designed for depression [52, 62, 67], or pregnant women [58, 69].

Intervention duration varied from 4 to 12 weeks (median: 8.5 weeks), frequency from 1 to 7 times per week (median: 2), and overall contact time with therapist from 2 to 72 h (median: 18 h). In 16 studies, yoga was conducted in groups [48, 49, 52, 54–58, 61, 63, 66, 68–72]. Homework was intended in 12 studies [48, 50, 52, 55–57, 65, 66, 68–71]. One intervention consisted of homework only [64].

Regarding cointerventions (psychotherapy and antidepressant medication), patients most commonly had to be on a stable dose before and during the intervention [48, 50, 52, 54–57, 61, 64–67, 70]. Other studies defined the usage of cointervention as an inclusion criterion [63], did not allow any cointervention [49, 59, 68, 72], or defined no restriction [71].

Twelve studies each had a passive [52, 54–58, 61–63, 66, 67, 71] and active control group [48, 49, 56–59, 61, 64, 65, 68–70]. Six studies were three-armed, with a passive and an active control group [56–59, 61, 62]. One each compared different dosages [50, 51] and components [72] of yoga intervention.

#### 3.2.3. Outcome Measures

Severity of depression mostly was measured with the Hamilton Depression Rating Scale (HDRS) [52, 57, 59, 61–63, 65–67, 71] and the Beck Depression Inventory (BDI) [49, 54, 56, 59, 64, 66, 68, 72]. Remission was defined as a cutoff score of depression severity by nine studies [49, 50, 54, 59, 65–68, 70], two studies used diagnostic criteria [57, 71], and one combination of both [61]. One study did not report its definition of remission [52]. Quality of life was assessed by the SF-12 questionnaire [48, 55], subscales of the SF-20 [70], or items of the WHO QoL-BREF [71]. Safety was assessed by reporting side effects [59], adverse events [50, 56, 66, 68], serious adverse events [70, 71], or their combination [49, 61, 69].

### 3.3. Risk of Bias in Individual Studies

In four studies, per protocol analysis was conducted [57, 62, 63, 67]. High dropout rates led to a high risk of bias in missing outcome data in six studies [49, 52, 55, 58, 63, 67]. Because of the nature of yoga and the measurement of a subjective outcome, blinding of participants and outcome assessment was not possible. The risk of bias for measurement of the outcome, therefore, is always rated as “some concerns” (active control) or “high” (passive control) except for one study comparing different interventions, where participants were blinded to group allocation [72]. Due to the fact that overall bias represents the highest score of subdomains, an overall bias of “low” cannot be reached. We therefore decided to omit reporting of overall bias (Supporting Information 2).

### 3.4. Analyses of Overall Effect

#### 3.4.1. Depression Severity

**Figure 2:**
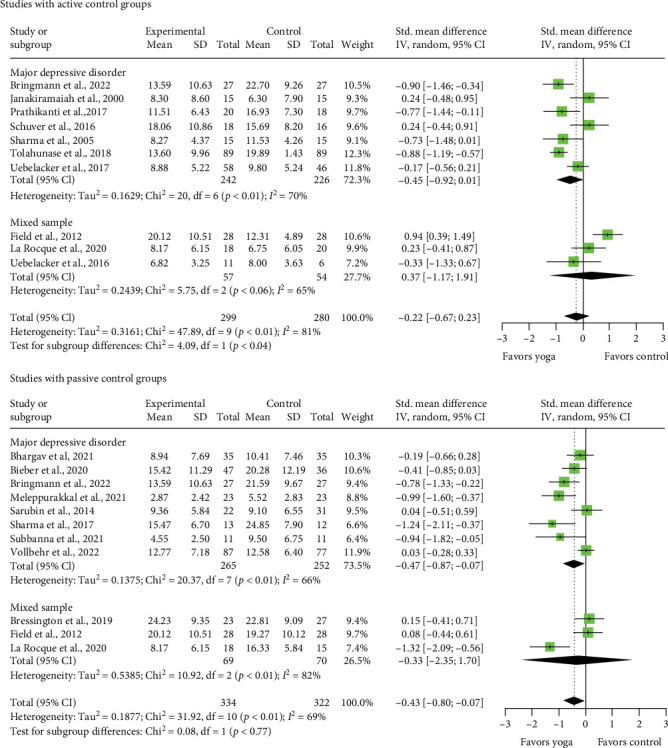
Forest plot for depression severity. CI, confidence interval.

Compared to passive control, yoga interventions showed a statistically significant short-term effect on depression severity (SMD = −0.43, 95% CI = [−0.80; −0.07]; MD = −5.05, 95% CI = [9.40; −0.82]; CLES = 61.95 %). In subgroup analysis, a statistically significant effect accounted for studies including patients with MDD (SMD = −0.47, 95% CI = [−0.87; −0.07]; MD  = 5.52, 95% CI = [−10.22; −0.82]; CLES = 63.02%; 8 RCTs), but not for mixed samples (SMD = −0.33, 95% CI = [−2.35; 1.70]; MD = −3.88, 95% CI = [−27.61; 19.98]; CLES = 59.23%; 3 RCTs). None of the effects reached clinical relevance of 6.45 points. Compared to active control groups, yoga interventions showed no statistically significant short-term effect on depression severity (SMD = −0.22, 95% CI = [−0.67; 0.23]; MD = 2.59, 95% CI = [7.87; 2.70]; CLES = 56.18%). In subgroup analysis, no statistically significant effects occur (MDD: SMD  = −0.45, 95%CI = [−0.92; 0.01], MD = 5.29, 95% CI = [10.81; 0.12], CLES = 62.48%, 7 RCTs; mixed samples: SMD = 0.37,95%CI = [−1.17; 1.91], MD = 4.35, 95% CI = [−13.75;22.44], CLES = 39.68%, 3 RCTs) (Figure 2). Heterogeneity was substantial for passive and considerable for active control (Figure 2), quality of evidence was rated very low for passive and active control (Supporting Information 3).

Regarding intermediate and long-term effects, compared to active control, no statistically significant group differences were observed in two studies (one with MDD and mixed samples each) [57, 64], while two studies showed statistically significantly greater improvements of depression severity in the intervention group after 1 year in a mixed sample [48] and 3 months postintervention (PI) in an MDD sample [70]. Two studies with passive control, including MDD [71] and mixed samples [57], showed no statistically significant difference between intervention groups in long and intermediate-term outcomes.

#### 3.4.2. Remission

Compared to passive control, yoga participants showed a statistically significant higher remission rate (OR = 3.20; 95% CI = [1.45; 7.10]; CLES = 67.49%). Both subgroups showed statistically significant effects (MDD: OR = 2.28, 95% CI = [1.25; 4.16], CLES = 62.6%, 4 RCTs; mixed samples: OR = 22.00, 95% CI = [2.34; 206.48], CLES = 88.59%, 1 RCT).

A statistically significant higher remission rate for yoga was also found compared to active control (OR = 2.04; 95% CI = [1.13; 3.69]; CLES = 60.95%). In the subgroup of MDD, effects were statistically significant (OR = 2.31; 95% CI = [1.17; 4.55]; CLES = 62.79%; 5 RCTs), while mixed samples showed no statistically significant effect (OR  = 1.05; 95% CI  = [0.28; 3.86]; CLES  = 50.76%; 1 RCT).

**Figure 3:**
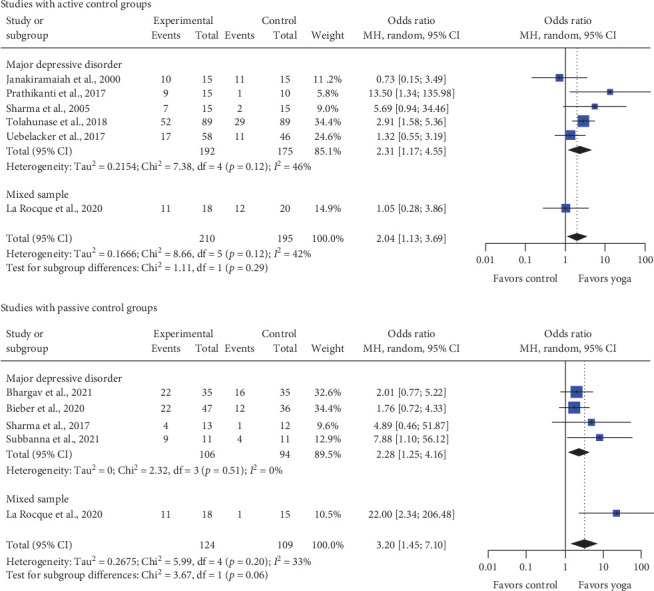
Forest plot for remission rate. CI, confidence interval.

Heterogeneity was moderate, and the quality of evidence was moderate for passive and low for active control (Figure 3, Supporting Information 3).

For intermediate and long-term effects, one study with active control showed no statistically significant difference in an MDD sample [70], while in a mixed sample with active control, statistically significantly more participants of yoga intervention experienced remission than control group 9 months PI [57].

#### 3.4.3. Quality of Life

When compared to passive control in a mixed sample, mental health-related quality of life improved statistically significant in the short-term, but no difference was observed after 3 months [55]. Another study with passive control revealed a statistically significant improvement in quality of life for both groups after 12 months follow-up [71]. A study with active control and mixed sample showed improvement in mental health-related quality of life for both groups with no group difference after 1 year follow-up[48].

#### 3.4.4. Safety

**Figure 4:**
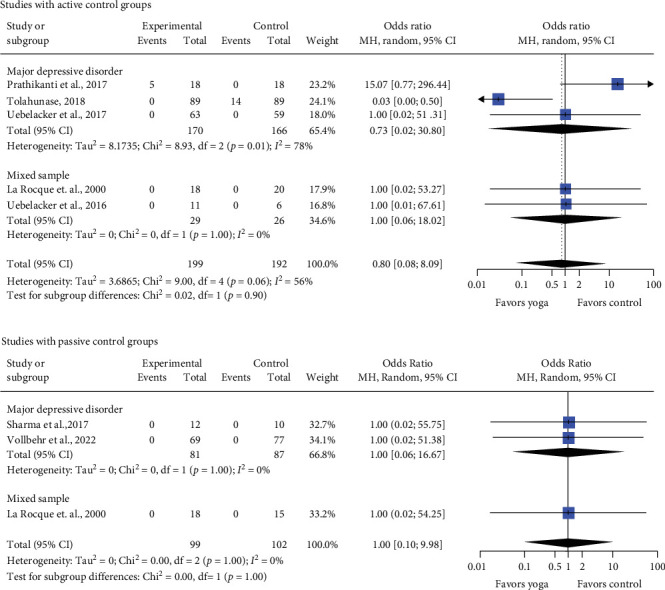
Forest plot for safety. CI, confidence interval.

Meta-analysis showed no statistically significant differences between yoga interventions and passive control (OR = 1.00, 95% CI = [0.10; 9.98], CLES = 50.00%; MDD: OR = 1.00, 95% CI = [0.06; 16.67], CLES = 50.00%, 2 RCTs; mixed samples: OR = 1.00, 95% CI = [0.02; 54.25], CLES = 50.00%, 1 RCT). Also for active control no statistically significant differences on safety outcomes was found (OR = 0.80, 95% CI = [0.08; 8.09], CLES = 46.53%; MDD: OR = 0.73, 95% CI = [0.02; 30.80], CLES = 45.12%, 3 RCTs; mixed samples: OR = 1.00, 95% CI = [0.06; 18.02], CLES = 50.00%, 2 RCTs). Heterogeneity is low for passive and substantial for active control groups (Figure 4). The quality of evidence was rated as very low (Supporting Information 3).

### 3.5. Studies not Included in Meta-Analysis

Comparing two Sudarshan Kriya Yoga interventions, one including cyclical breathing and one with normal breathing, both groups showed an improvement in depression severity with no statistically significant group difference [72]. Another study compared two different dosing groups of yoga intervention and found no statistically significant group differences in depression severity change. Increasing practicing time is inversely correlated with severity scores [50, 51].

### 3.6. Sensitivity Analyses

Sensitivity analysis regarding the risk of bias was not conducted (see Section 3.3). No subgroup analysis could be performed for studies that included only women, as the respective subgroups included fewer than two studies.

### 3.7. Risk of Bias Across Studies

Regarding passive controls, the funnel plot showed statistically significant asymmetry (Egger's test: *p*=0.02), indicating publication bias toward studies favoring yoga. For studies with active control, the funnel plot indicated no publication bias (Egger's test: *p*=0.28) (Supporting Information 4).

## 4. Discussion

### 4.1. Summary of Evidence

Our meta-analysis found a statistically significant small-size effect of yoga interventions on depression severity when compared to passive but not to active controls. The quality of evidence was rated very low, and effect sizes missed clinical relevance. Yoga interventions were associated with statistically significant higher remission rates compared to passive and to active controls. The quality of evidence was moderate for passive and low for active controls. Effects on quality of life were quite heterogeneous. Yoga interventions can be considered safe, with very low-quality evidence.

### 4.2. Agreement With Prior Systematic Research

A positive effect of yoga intervention on depression severity was also observed in prior metaanalyses and reviews [25, 29, 73, 74], partly even when compared to active control [24, 26, 27]. One meta-analysis showed no statistically significant effect on depression severity, probably due to the high heterogeneity of included studies [75].

In our meta-analysis, all participants had a clinical diagnosis of depressive disorder. In a recent analysis, clinical diagnosis of depression was shown to be a potential effect moderator, being associated with a higher effect of yoga intervention [29]. However, another meta-analysis showed no effect on severity for patients with clinical diagnosis but limited evidence for patients with elevated levels of depression when comparing yoga to usual care [27].

In a study comparing two yoga interventions with and without cyclical breathing, no statistically significant difference between groups appeared [72]. A dose–response study showed a correlation of higher cumulative time of yoga practice with a decrease in depression scores [50, 51]. Earlier research showed that interventions including meditation and relaxation [27, 29], of longer duration [29] and higher frequency [73] have greater effectiveness.

### 4.3. External and Internal Validity

The included studies represent a middle-aged, predominantly white sample. The majority of studies originated from North America and Europe; approximately one-third come from Asia. Samples were predominantly female. Since women are more affected by depression than men [76], study samples can overall be considered quite representative, especially for Western countries. Clinical diagnosis of depression as an eligibility criteria leads to a high internal validity.

A publication bias regarding studies with passive control favoring the control group has to be considered when interpreting results.

### 4.4. Strengths and Limitations

This analysis is based on a systematic literature search and a clear definition of inclusion criteria, resulting in high internal validity. With 24 included RCTs, the review represents a relatively large body of evidence. Analyses further allow distinguishing between different control groups and diagnoses.

Given the nature of yoga interventions and patient-reported outcome measurements, blinding outcome assessors to group allocation is not possible. Reporting and expectation bias must be taken into account. Reporting of data regarding quality of life and safety of interventions is very heterogeneous. Adverse events probably were not assessed systematically, therefore safety-related results must be interpreted carefully. Control groups were separated into active and passive. Although most passive control groups consist of waitlist or treatment as usual (TAU), certain heterogeneity should be pointed out, particularly for active controls. Further subdivision could not be conducted for resulting in too small subgroups.

Other limitations concern methodological quality and reporting of the included RCTs. The heterogeneity of yoga interventions is high, so that no conclusion regarding the effectiveness of different types of interventions can be drawn.

### 4.5. Implication for Further Research

In order to evaluate which aspects and types of yoga are most effective for treating depression, research with more standardized interventions is necessary. Systematic comparison of different frequencies and durations of yoga interventions would allow to define the most effective timeframes. A general definition for remission of depression, as well as more standardized reporting of adverse events and quality of life, would improve the ability to draw conclusions regarding these outcomes.

### 4.6. Implication for Clinical Practice

Yoga represents a safe therapeutic approach showing positive results for depression severity when compared to passive control and higher remission rates than passive and active controls. Therefore, and in light of the absence of serious negative effects and the high number of patients not responding to standard therapy, yoga should always be considered as a possible therapeutic option, particularly for MDD patients.

## 5. Conclusion

Our meta-analysis provides evidence for yoga as a safe therapeutic option for patients with depressive disorders, with statistically significant small-size effects on depression severity compared to passive control and statistically significant higher remission rates compared to passive and active controls. The quality of evidence ranges from very low to moderate. Further research is needed to evaluate longterm effects and the most effective type and timeframe of the intervention.

## Figures and Tables

**Table 1:** Characteristics of included studies.

References	Origin	Sample characteristics	Sample size		Recruiting	Cointervention	Intervention		Measure timepoints	Outcome measures (1) severity (2) remission (3) QoL (4) safety
			Intervention	Control			Treatment	Control		
Bhargav et al. [52]	Asia (India)	70 Participants with MDD (DSM-IV) and HDRS ≥18 31.5 ± 8.7 years, gender and ethnicity not reported	35	35	At tertiary care center	No ECT in the last 3 months, antidepressants stabilized for at least 2 weeks	Validated yoga module for depression by trained yoga instructor, including poses, breathing, yoga counseling [53] in groups, 60 min, 4x/week for 8 weeks, homework and two group booster sessions in week 5–12	Waitlist	Baseline, week 4, week 8, week 12 (PI)	(1) HDRS-17 (2) Unknown definition

Bieber et al. [54]	Europe (Germany)	83 Participants (outpatients) with MDD (DSM-IV) and BDI ≥14 49.7 ± 9.8 years, 20.5% female, 90.4% German	47	36	Via outpatient clinic	No initiation of psychotherapy during or 2 months before intervention; both groups received psychopharmacological and psychotherapy (TAU)	Ashtanga Yoga by licensed yoga instructor, including poses, breathing, meditation in groups of 10–12, 90 min, 3x/week for 12 weeks	Waitlist	Baseline, week 6, week 12	(1) BDI-II (2) BDI—II ≤12

Bressington et al. [55]	Asia (China)	50 Participants (community dwelling people) with MDD or non-MDD (ICD-10) and DASS ≥ 10 YG: 46.3 ± 12.8 years, CG: 49.4 ± 9.13 years 70% female, ethnicity not reported	23	27	In community via nurse consultant	Use of antidepressants and no plan to change during next 3 months as condition, TAU continued during intervention (medication and outpatient appointments)	Laughter Yoga by certified trainer and coinvestigator, including breathing and laughter meditation in groups, 45 min, 2x/week for 4 weeks homework encouraged (workbook)	TAU: routine community mental health care, medications, and outpatient appointments	Baseline, week 4 (PI), week 12 (FU)	(1) DASS-21 (3) SF-12

Bringmann et al. [56]	Europe (Germany)	81 Participants (outpatients) with mild to moderate depression (ICD-10) and BDI-II ≥ 10 MBLM: 49.1 ± 11.1 years, Control: 51.0 ± 12.7 years, TAU: 45.0 ± 11.1 years 80.2% female ethnicity not reported	27	TAU: 27 Control: 27	Via attending psychiatrist, flyers and posters	Antidepressant medication continued	Meditation-based lifestyle modification, based on classical yoga, by therapist, including ethical living, breathing, postures, meditation in groups 180 min, 1x/week for 8 weeks daily homework of 45 min	(1) TAU: individual multimodal therapy, including pharmacotherapy, psychotherapy, and accessory therapy (2) Control: drug therapy only	Baseline, week 4, week 8, 6 months (after rerandomization)	(1) BDI-II (4) AE

Butler et al. [57]	North America (USA)	52 Participants with MDD or non-MDD (DSM-IV) for at least 2 years without remission of ≥2 month 50.4 ± 14.8 years, 74% female, 87% Caucasian/White 9% Asian/Asian American, 2% Hispanic, 2% Middle Eastern, 2% American Native/Alaska Native, 9% other	15	Hypnosis: 15 Psychoeducation: 16	Internet advertisements, posted flyers, mailings to local physicians and mental health professionals and organizations	Stable dose of antidepressants for at least 3 months	Hatha Yoga with meditation by clinical psychologist and student co-leader, including meditation, postures, breathing, mantra in groups, 120 min 1 x/week for 8 weeks, 4 h retreat and booster session in week 12 homework encouraged for 30 min 6x/week; psychoeducational materials	(1) Hypnosis by psychiatrist or clinical psychology graduate student 90 min, 1x/week, 10 weeks, booster session (120 min) in week 12, homework encouraged (techniques for self-hypnosis taught); psychoeducational materials (2) Psychoeducational materials	Baseline, 6 months, 9 months	(1) HDRS (26 item version) (2) No mood disorder (SCID) for ≥ 2 months

Field et al. [58]	North America (USA)	84 Pregnant women (18–22 weeks) with MDD or non-MDD (DSM-IV) 26.6 years, 48% Hispanic, 40% African American, 12% non-Hispanic white	28	Massage: 28 Control: 28	Screening at medical school prenatal ultrasound clinics	95% are not taking antidepressant medication or are receiving psychotherapy	Yoga routine designed for second and third-trimester pregnant women by trained instructor, including postures 20 min, 2x/week for 8 weeks	(1) Standard prenatal care (2) Full body massage, same duration and frequency as yoga intervention	Baseline, week 12 (PI)	(1) CES-D

Janakiramaiah et al. [59]	Asia (India)	45 Inpatients with melancholic depression (DSM-IV) and HDRS ≥ 17, YG: 36.0 ± 7.8 ECT: 36.7 ± 2.5 IMN: 43.4 ± 11.9 44.4% female, ethnicity not reported	15	ECT: 15 IMN: 15	Inclusion of consenting inpatients	Current episode not treated no psychotropic drugs allowed (except for IMN intervention)	Sudarshan Kriya Yoga, including breathing by well-trained art-of-living yoga teacher, 45 min 4–6x/week, 4 weeks	(1) IMN: 150 mg daily dose at night (2) ECT: 4 weeks, 3x/week (not included)	Baseline, week 1, week 2, week 3, week 4 (PI)	(1) BDI und HDRS-17 (2) HDRS-17 < 8

Kinser et al. [60] Kinser, Elswick, and Kornstein [48]	North America (USA)	27 Women with MDD or dysthymia (DSM-IV) and PHQ-9 ≥ 10 43.3 ± 15.6 years, 63.0% white non-Hispanic, 37.0% non-White	15	12	Material posted in public locations, offices of primary care providers, women's health providers, and mental health care providers	Usual depression care continued, no changes in antidepressant medication during last month	Hatha Yoga, including poses, breathing, relaxation, and meditation in groups by certified yoga teacher familiar with teaching yoga-naive students 75 min, 1x/week, 8 weeks daily homework of 20 min encouraged	Attention control group: health education by registered, trained nurses 75 min, 1x/week, 8 weeks handouts for reviewing at home	Baseline, week 2, week 4, week 6, week 8 (PI), week 52 (FU)	(1) PHQ-9 (3) SF-12 mental component (4) Suicidal ideation (PHQ-9)

La Rocque et al. [61]	North America (Canada)	53 Women with depressive disorder (DSM-V), YG: 34.17 ± 15.75 WL: 29.40 ± 13.08, EG: 34.85 ± 15.15, 73.58% white; 26.41% other	15	Waitlist: 15 Aerobic exercise: 20	Advertisement	No change in type or dose of medication or frequency of psychotherapy in previous 3 months	Bikram Yoga in temperature controlled room (~ 40°C), including breathing, poses, relaxation in groups by certified Bikram yoga teacher, 90 min, 2x/week, 8 weeks	(1) Aerobic exercise: 50–60 min, 2 x/week, 8 weeks group classes of cardio or strength training (2) Waitlist	Baseline, week 8 (PI)	(1) HDRS (2) Not meeting diagnosis criteria (SCID) and HDRS ≤ 7 (4) Minor AE, AE, SAE

Meleppurakkal, Sunitha, and Jayan [62]	Asia (India)	75 Participants with mild to moderate depression (ICD−10) 65–75 years, 54.7% female	25	Yoga group: 25 Churna group: 25	Screening of hospital patients	Inclusion irrespective to medication history cointervention during study not reported	Selected yoga techniques, including prayer, loosening exercises, poses, breathing, and relaxation for 30 days intake of 4 g churna 2x/day	(1) Churna only (2) yoga only	Baseline, 30 days (PI), 45 days (FU)	(1) HDRS

Prathikanti et al. [49]	North America (USA)	38 Participants with MDD (MINI) and BDI 14–28 43.4 ± 14.7 years, 68% female, 58% European, 16% Asian, 11% Latino, 8% African and 8% multi ethnic	20	18	Ads in libraries, community centers, shopping areas, online classifieds, and UCSF outpatient clinics and clinical trials websites	Antidepressant medication or psychotherapy not allowed (within 2 months of screening)	Hatha Yoga, including breathing, postures, relaxation in groups by certified yoga teacher and licensed, registered nurse, 90 min, 2x/week, 8 weeks	Yoga history modules by registered yoga teacher with additional certification as a Vedic Master Educator in yoga philosophy, 16x 90 min	Baseline, week 2, week 4, week 6, week 8 (PI)	(1) BDI-II (2) BDI ≤ 9 (4) SAE and AE

Sarubin et al. [63]	Europe (Germany)	53 Participants with MDD (DSM-IV), 40.25 ± 12.57 years, 28.3% female, ethnicity not reported	22	31	Not specified	During the intervention all participants received either QXR (300 mg/day) or ESC (10 mg/day)	Hatha Yoga in groups by physical therapist, 60 min, 1x/week, 5 weeks	Medication only	Days 1, 4, 7, weeks 2, 3, 4, 5	(1) HDRS-21

Schuver and Lewis [64]	North America (USA)	40 Participants with MDD (DSM-IV) and BDI ≥ 14 42.7 ± 4.95 years, 100% female, 95% non-Hispanic	20	20	Targeted email via local newsletter, Craigs List	No change in forms of other treatments (antidepressant medication, psychotherapy) during previous month or during intervention period	Mindfulness-based yoga condition, including poses, breathing, meditation, practiced at home, 60–75 min, at least 2x/week, 12 weeks 8 x 15 min mindfulness-based telephone session	Walking-based workout via DVD or other walking at least 65 min, 2x/week, 12 weeks 8x 15 min telephone session discussing a weekly health-related topic	Baseline, week 12 (PI), week 16 (FU)	(1) BDI

Sharma et al. [65]	Asia (India)	30 Participants (outpatients) with MDD (DSM-IV) who have not been treated for current depressive episode YG: 31.9 years, CG: 31.7 years, 31% female, ethnicity not reported	15	15	Hospital outpatient services of department of psychiatry	Conventional antidepressant treatment in both groups	Sahaj Yoga, including meditation by a trained yoga instructor, setting not specified 30 min, 3x/week, 8 weeks homework encouraged	Same environment and attention provided without actual meditation practice	Baseline, week 8 (PI)	(1) HDRS-17 (2) HDRS-17 ≤ 7

Sharma et al. [66]	North America (USA)	25 Outpatients with MDD (DSM-IV), inadequate response to antidepressants (intake ≥ 8 weeks) and HDRS−17 ≥ 14 YG: 39.4 ± 13.9 years, WG: 34.8 ± 13.6 years, 72% female, 92% Caucasian, 8% African-American	13	12	Outpatients enrolled in the University of Pennsylvania Mood and Anxiety Disorders Treatment and Research Program (MADTRP)	≥8 weeks stable dose of antidepressants, no change during intervention	Sudarshan Kriya Yoga, including poses, breathing, meditation by certified instructor in group 210 min 6x/week in week 1, 90 min 1x/week in week 2–8 homework: 20–25 min daily in weeks 2–8	Waitlist	Baseline, week 4, week 8 (PI)	(1) HDRS-17, BDI (2) HDRS-17 ≤ 7 and reduction > 50% (4) TEAE

Subbana et al. [67]	Asia (India)	36 Participants with MDD (DSM-IV) and HAMD ≥ 18 demographic data not further specified	18	18	Not reported	≥ 2 Weeks stable dose of antidepressants, both groups receive TAU	Generic yoga module developed for people with depression, 60 min, 4 – 6x/week, 12 weeks	Waitlist (TAU)	Baseline, week 4, week 8, week 12 (PI)	(1) HDRS, MADRS, CGI (2) HDRS < 7

Tolahunase, Sagar, and Dada [68]	Asia (India)	178 outpatients with MDD (DSM-IV) and BDI <50 YG: 38 ± 9 years, DG: 40 ± 8 years 47.8% female	89	89	Via Psychiatry Department of Institute Hospital	Patients in the YG were asked to stop antidepressant medication and did not receive antidepressant medication during trail	Mix of Hatha and Raja Yoga, including postures, breathing, and meditation by registered, specialized yoga instructor 120 min, 5x/week, 12 weeks week 1−2: groups, including interactive lectures of yoga, lifestyle, and health, week 3–12: one-on-one and unsupervised sessions	Drug therapy: SSRI as described by treating psychiatrist	Baseline, week 12 (PI)	(1) BDI-II (2) BDI-II ≤ 9 (4) AE and minor AE

Uebelacker et al. [69]	North America (USA)	20 Pregnant women (12–26 weeks) with MDD or non-MDD (DSM-IV) and QIDS 7– 20, 28.4 ± 5.8 years, 5% Latina, 95% non-Latina	12	8	Advertising at OB/GYN and other community locations	Not specified	Prenatal yoga program, including breathing, meditation, poses in group by registered yoga instructor, 75 min, 1x/week, 9 weeks, homework encouraged	Mom–baby wellness workshop: 75 min 1x/week, addressing mother's and baby's health and wellness by master's level instructors with prior training in psychology and study-specific training in perinatal health	Baseline, week 3, week 6, week 9	(1) QIDS (2) AE and SAE

Uebelacker et al. [70]	North America (USA)	122 Participants with MDD (DSM-IV) and QIDS of 8–17 46.5 ± 12.2 years, 84.4% female 84.4% white or Caucasian, 3.3% Black or African American, 12,3% other or multiracial	63	59	Via advertisement in community	Current intake of antidepressant medication with dose of demonstrated effectiveness for at least 8 weeks and stable for ≥ 4 weeks, psychotherapy frequency stable for ≥ 6 weeks, both no plans to change in next 10 weeks	20–30 min individual introductory meeting, Hatha yoga, including postures, meditation, breathing, and relaxation by registered yoga teacher with yoga alliance 80 min, 1–2x/week for 10 weeks, homework encouraged	Healthy living workshop: initial individual orientation meeting, 60 min, 1–2x/week, 10 weeks	Baseline 1 (eligibility), baseline 2 (randomization), 3.3 weeks, 6.6 weeks, 10 weeks (PI), 3 months (after PI), 6 months (after PI)	(1) QIDS (2) QIDS ≤ 5 (3) SF-20 subscales (4) SAE

Vollbehr et al. [71]	Europe (Netherlands)	171 Outpatients, women between 18 and 34 years with MDD (DSM-IV) YG: 25.2 ± 4.9 years, TAU: 24.9 ± 4.4 years	88	83	Outpatients in psychiatry clinics	TAU, no restriction in changing during intervention except of adding an extra intervention for control group to compensate not receiving yoga	Mindful-yoga-intervention: Hatha yoga and instructions to increase mindful awareness by trained yoga teacher in groups 90 min, 1x/week, 9 weeks homework of 30 −45 min on other days encouraged, videos provided	TAU: individualized standard care administered according to Dutch treatment guidelines by health professional	Baseline, week 10–15 (PI), 6 month (FU), 12 month (FU)	(1) HDRS-17, DASS (2) SCID (3) WHOQOL-BREF (4) SAE

Included for qualitative analysis										

Rohini et al. [72]	Asia (India)	30 Inpatients with MDD (DSM-IV) and HDRS ≥18 SKY: 29.5 ± 8.2 years, partial SKY: 34.2 ± 11.7 years 53.3% female	15	15	Inpatients attending psychiatric services of NIMHANS	Drug-naive or off medication for at least 4 weeks, only lorazepam or zopiclone during intervention if needed	Sudarshan Kriya Yoga including Ujjai, Bhastrika, cyclical breathing, yoganidra by yoga teacher in group 7x/week, 4 weeks	Partial Sudarshan Kriya Yoga: regular breathing instead of cyclical breathing component, same frequency and duration	Baseline, week 1, week 2, week 3, week 4	(1) BDI

Streeter et al. [50] and Scott et al. [51]	North America (USA)	32 Participants with MDD (DSM-IV) and BDI ≥14 HDG: 38.4 ± 15.1 years, LDG: 34.7 ± 10.4 years, 83.3% female	16	16	Internet and advertisement	Stable dose of antidepressant for at least 3 months without dose change during intervention	HDG: Yoga including postures, relaxation, breathing by Iyengar Introductory level II certification group sessions of 90 min 3x/week and homework of 30 min 4x/week, 12 weeks	LDG: same intervention, but reduced to group sessions 2x/week and homework 3x/week	Baseline, week 4, week 8, week 12 (PI)	(1) BDI-II, PHQ-9 (2) BDI-II < 14 (4) AE

Abbreviations: AE, adverse event; BDI, Beck Depression Inventory; CES-D, Center for Epidemiologic Studies Depression Scale; CG, control group; DASS, Depression Anxiety Stress Scale; DG, drug group; DSM-IV, diagnostic and statistical manual of mental disorders; ECT, electroconvulsive therapy; EG, exercise group; FU, follow-up; HDG, high dose group; HDRS (also HAM-D or HRSD), Hamilton Depression Scale; ICD-10, International Classification of Disease; IMN, imipramine; LDG, low dose group; MBLM, meditation-based lifestyle modification; MDD, major depressive disorder; MINI, mini international neuropsychiatric interview; PHQ-9, patient health questionnaire; PI, postintervention; QIDS, quick inventory of depressive symptomatology; SAE, severe adverse event; SCID, structured clinical interview for DSM-V; SF-12/20, short form-12/20; SKY, Sudarshan Kriya Yoga; TAU, treatment as usual; TEAE, treatment-emergent adverse event; WG, waitlist group; YG, yoga group.

## Data Availability

The data supporting this meta-analysis are from previously reported studies and datasets, which have been cited. The processed data are available on the open science framework.

## Supporting Information

Supporting Information Online only supplements are provided. These include. *Supporting Information 1*. Search strategy for systematic literature search. *Supporting Information 2*. Risk of bias in individual studies. *Supporting Information 3*. GRADE rating for quality of evidence. *Supporting Information 4*. Funnel plots for studies reporting depression severity with active and passive controls. *Supporting Information 5*. PRISMA 2020 Checklist.
