# Supplementary material for: Yoga for Depressive Disorder: A Systematic Review and Meta-Analysis
Source: Depress Anxiety. 2024 Dec 19;2024:6071055. doi: 10.1155/da/6071055 (PMC11919030; doi:10.1155/da/6071055)
Supplement: Supporting Information — Online only supplements are provided. These include. Supporting Information 1. Search strategy for systematic literature search. Supporting Information 2. Risk of bias in individual studies. Supporting Information 3. GRADE rating for quality of evidence. Supporting Information 4. Funnel plots for studies reporting depression severity with active and passive controls. Supporting Information 5. PRISMA 2020 Checklist. [file 6071055.f1.pdf]

Supplementary 1: Search strategy for systematic literature search

Supplementary 2: Risk of bias in individual studies

Supplementary 3: GRADE rating for quality of evidence

Supplementary 4: Funnel plots for studies reporting depression severity with active and passive controls

Supplementary 5: PRISMA 2020 Checklist

Supplementary 1: Search strategy for systematic literature search

PubMed:

(“Depression”[Mesh] OR “Depressive Disorder”[Mesh] OR depress* [Title/Abstract] OR dysthymi*[Title/ Abstract]) AND (“Yoga”[Mesh] OR *yoga[Title/Abstract] OR yoga* [Title/Abstract] OR yogi* [Title/Abstract] OR asana* [Title/Abstract] OR pranayama [Title/Abstract] OR dhyana [Title/Abstract] OR Surya Namaskara [Title/Abstract])

Cochrane:

((depress*.ti,ab,kw,sh) OR (dysthmi*.ti,ab,kw)) AND ((yoga*.ti,ab,kw,sh) OR (yogi*.ti,ab,kw) OR (asana*.ti,ab,kw) OR (pranayama.ti,ab,kw) OR ("Surya Namaskara".ti,ab,kw) OR (dhyana.ti,ab,kw))

Scopus:

TITLE-ABS-KEY (*yoga) OR TITLE-ABS-KEY (yoga*) OR TITLE-ABS-KEY (yogi*) OR TITLE-ABS-KEY (asana*) OR TITLE-ABS-KEY (pranayama) OR TITLE-ABS-KEY (dhyana) OR TITLE-ABS-KEY ("surya namaskara") AND (TITLE-ABS-KEY (depress*) OR TITLE-ABS-KEY (depressive AND disorder) OR TITLE-ABS-KEY (dysthymi)) AND NOT TITLE-ABS-KEY (animal OR tissue OR mice OR cancer OR review OR observational OR "case report" OR "case study" OR parkinson OR sclerosis OR *arthritis OR covid-19 OR cardiac OR infant OR "chronic pain" OR "back pain" OR hyperten* OR burnout) AND (LIMIT-TO (SRCTYPE , "j")) AND (LIMIT-TO (DOCTYPE , "ar"))

PsyInfo:

((Any Field: (*yoga)) OR Any Field: (*yoga) OR (Any Field: (yogi*)) OR (Any Field: (asana*)) OR (Any Field: (pranayama)) OR (Any Field: (dhyana)) OR (Any Field: (surya namaskara))) AND ((Any Field: (depress*)) OR (Any Field: (dysthm*)))

	Comparator	D1	D2	D3	D4	D5
Bhargav et al., 2021	passive	+	+	-	-	!
Bieber et al., 2021	passive	!	+	+	-	-
Bressington et al., 2019	passive	!	!	-	-	!
Bringmann et al., 2022	active and passive	!	!	+	-	+
Butler et al., 2008	active and passive	+	!	+	-	+
Field et al., 2012	active and passive	!	!	!	-	!
Janakiramaiah et al., 2000	active	!	+	+	!	!
Kinser et al., 2013/14	active	!	-	!	!	+
La Rocque et al., 2021	active and passive	+	+	+	-	+
Meleppurakkal et al., 2021	passive	!	!	+	-	!
Prathikanti et al., 2017	active	+	+	-	!	+
Rohini et al., 2000	dose - response	!	+	+	+	!
Sarubin et al., 2020	passive	!	-	-	-	+
Schuver and Lewis, 2016	active	!	-	-	!	+
Sharma et al., 2005	active	!	+	+	!	+
Sharma et al., 2017	passive	!	+	+	-	+
Streeter et al., 2017, Scott et al.. 2019	dose - response	+	!	+	!	!
Subbana et al., 2021	passive	!	!	-	-	+
Tolahunase et al., 2018	active	+	+	+	!	+
Uebelacker et al., 2016	active	!	+	+	!	!
Uebelacker et al., 2017	active	+	+	+	!	+
Vollbehr et al., 2022	passive	+	+	+	-	+

+ Low risk
! Some concerns
- High risk

D1 Randomisation process
D2 Deviations from the intended interventions
D3 Missing outcome data
D4 Measurement of the outcome
D5 Selection of the reported result

Supplementary 2: Risk of bias in individual studies

Summary of findings:

Yoga compared to active control for depression

Patient or population: depression
Setting:
Intervention: Yoga
Comparison: active control

Outcomes	Anticipated absolute effects* (95% CI)		Relative effect (95% CI)	№ of participants (studies)	Certainty of the evidence (GRADE)	Comments
	Risk with active control	Risk with Yoga				
severity	-	SMD **0.22 SD lower** (0.62 lower to 0.18 higher)	-	579 (10 RCTs)	⊕○○○ Very low[a,b,c]	
remission	338 per 1.000	**507 per 1.000** (368 to 644)	**OR 2.01** (1.14 to 3.54)	405 (6 RCTs)	⊕⊕○○ Low[b]	
safety	73 per 1.000	**59 per 1.000** (6 to 389)	**OR 0.80** (0.08 to 8.09)	391 (5 RCTs)	⊕○○○ Very low[b,d,e]	

***The risk in the intervention group** (and its 95% confidence interval) is based on the assumed risk in the comparison group and the **relative effect** of the intervention (and its 95% CI).

CI: confidence interval; **OR:** odds ratio; **SMD:** standardised mean difference

GRADE Working Group grades of evidence
High certainty: we are very confident that the true effect lies close to that of the estimate of the effect.
Moderate certainty: we are moderately confident in the effect estimate: the true effect is likely to be close to the estimate of the effect, but there is a possibility that it is substantially different.
Low certainty: our confidence in the effect estimate is limited: the true effect may be substantially different from the estimate of the effect.
Very low certainty: we have very little confidence in the effect estimate: the true effect is likely to be substantially different from the estimate of effect.

Explanations

a. due to inconsistency
b. due to imprecision
c. due to dose response gradient
d. due to high risk of bias
e. due to possible confounding

Summary of findings:

Yoga compared to passive control for depression

Patient or population: depression
Setting:
Intervention: yoga
Comparison: passive control

Outcomes	Anticipated absolute effects* (95% CI)		Relative effect (95% CI)	№ of participants (studies)	Certainty of the evidence (GRADE)	Comments
	Risk with passive control	Risk with yoga				
severity	-	SMD **0.43 SD lower** (0.12 lower to 0.75 lower)	-	656 (11 RCTs)	⊕○○○ Very low[a,b,c,d,e]	
remission	312 per 1.000	**592 per 1.000** (397 to 763)	**OR 3.20** (1.45 to 7.10)	233 (5 RCTs)	⊕⊕⊕○ Moderate[a,c,f]	
saftey	0 per 1.000	**0 per 1.000** (0 to 0)	**OR 1.00** (0.10 to 9.98)	201 (3 RCTs)	⊕○○○ Very low[a,c,g]	

***The risk in the intervention group** (and its 95% confidence interval) is based on the assumed risk in the comparison group and the **relative effect** of the intervention (and its 95% CI).

CI: confidence interval; **OR:** odds ratio; **SMD:** standardised mean difference

GRADE Working Group grades of evidence
High certainty: we are very confident that the true effect lies close to that of the estimate of the effect.
Moderate certainty: we are moderately confident in the effect estimate: the true effect is likely to be close to the estimate of the effect, but there is a possibility that it is substantially different.
Low certainty: our confidence in the effect estimate is limited: the true effect may be substantially different from the estimate of the effect.
Very low certainty: we have very little confidence in the effect estimate: the true effect is likely to be substantially different from the estimate of effect.

Explanations

a. due to high risk of bias
b. due to inconsistency
c. due to imprecision
d. due to publication bias
e. due to dose response gradient
f. due to large effect
g. due to possible confounding

Supplementary 3: GRADE rating for quality of evidence

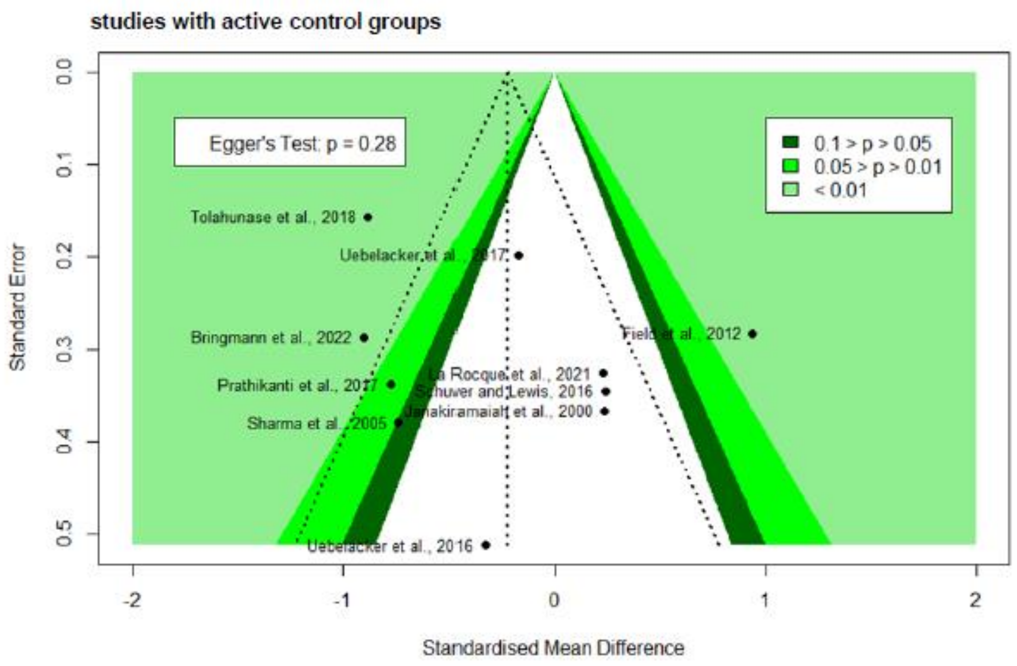

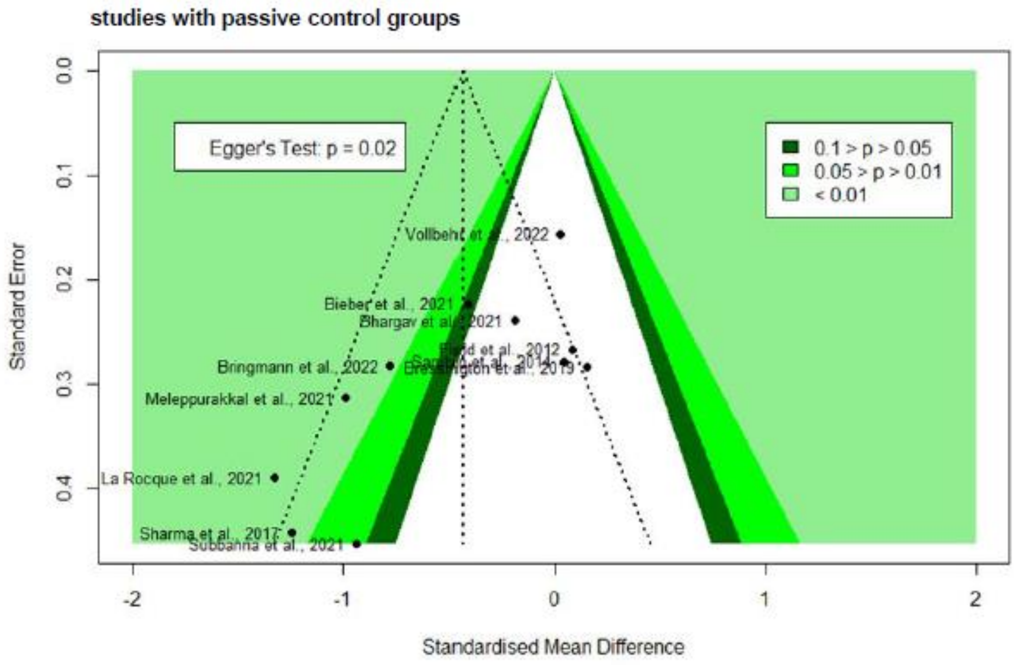

Supplementary 4: Funnel plots for studies reporting depression severity with active and passive controls

Supplementary 5: PRISMA 2020 Checklist

Section and Topic	Item #	Checklist item	Location where item is reported
**TITLE**			
Title	1	Identify the report as a systematic review.	title
**ABSTRACT**			
Abstract	2	See the PRISMA 2020 for Abstracts checklist.	abstract
**INTRODUCTION**			
Rationale	3	Describe the rationale for the review in the context of existing knowledge.	1. Introduction
Objectives	4	Provide an explicit statement of the objective(s) or question(s) the review addresses.	1. Introduction
**METHODS**			
Eligibility criteria	5	Specify the inclusion and exclusion criteria for the review and how studies were grouped for the syntheses.	2.1 Eligibility criteria
Information sources	6	Specify all databases, registers, websites, organisations, reference lists and other sources searched or consulted to identify studies. Specify the date when each source was last searched or consulted.	2.2 Search strategy
Search strategy	7	Present the full search strategies for all databases, registers and websites, including any filters and limits used.	eMethods
Selection process	8	Specify the methods used to decide whether a study met the inclusion criteria of the review, including how many reviewers screened each record and each report retrieved, whether they worked independently, and if applicable, details of automation tools used in the process.	2.2 Search strategy
Data collection process	9	Specify the methods used to collect data from reports, including how many reviewers collected data from each report, whether they worked independently, any processes for obtaining or confirming data from study investigators, and if applicable, details of automation tools used in the process.	2.3 Data extraction management
Data items	10a	List and define all outcomes for which data were sought. Specify whether all results that were compatible with each outcome domain in each study were sought (e.g. for all measures, time points, analyses), and if not, the methods used to decide which results to collect.	2.1.4 Outcome measures
	10b	List and define all other variables for which data were sought (e.g. participant and intervention characteristics, funding sources). Describe any assumptions made about any missing or unclear information.	2.3 Data extraction management, Table 1
Study risk of bias assessment	11	Specify the methods used to assess risk of bias in the included studies, including details of the tool(s) used, how many reviewers assessed each study and whether they worked independently, and if applicable, details of automation tools used in the process.	2.4 Risk of bias in individual studies
Effect measures	12	Specify for each outcome the effect measure(s) (e.g. risk ratio, mean difference) used in the synthesis or presentation of results.	2.5 Overall effect sizes

Section and Topic	Item #	Checklist item	Location where item is reported
Synthesis methods	13a	Describe the processes used to decide which studies were eligible for each synthesis (e.g. tabulating the study intervention characteristics and comparing against the planned groups for each synthesis (item #5)).	2.1.4 Outcome measures, 2.3 Data extraction management
	13b	Describe any methods required to prepare the data for presentation or synthesis, such as handling of missing summary statistics, or data conversions.	2.5 Overall effect sizes
	13c	Describe any methods used to tabulate or visually display results of individual studies and syntheses.	2.5 Overall effect sizes, Figure 2,3,4
	13d	Describe any methods used to synthesize results and provide a rationale for the choice(s). If meta-analysis was performed, describe the model(s), method(s) to identify the presence and extent of statistical heterogeneity, and software package(s) used.	2.5 Overall effect sizes
	13e	Describe any methods used to explore possible causes of heterogeneity among study results (e.g. subgroup analysis, meta-regression).	2.6 Subgroups, sensitivity analyses and meta-regression
	13f	Describe any sensitivity analyses conducted to assess robustness of the synthesized results.	2.6 Subgroups, sensitivity analyses and meta-regression
Reporting bias assessment	14	Describe any methods used to assess risk of bias due to missing results in a synthesis (arising from reporting biases).	2.9 Risk of bias across studies
Certainty assessment	15	Describe any methods used to assess certainty (or confidence) in the body of evidence for an outcome.	2.8 Quality of evidence
**RESULTS**			
Study selection	16a	Describe the results of the search and selection process, from the number of records identified in the search to the number of studies included in the review, ideally using a flow diagram.	3.1 Literature search, Figure 1
	16b	Cite studies that might appear to meet the inclusion criteria, but which were excluded, and explain why they were excluded.	3.1 Literature search
Study characteristics	17	Cite each included study and present its characteristics.	Table 1

Section and Topic	Item #	Checklist item	Location where item is reported
Risk of bias in studies	18	Present assessments of risk of bias for each included study.	3.3 Risk of bias in individual studies, eFigure 1
Results of individual studies	19	For all outcomes, present, for each study: (a) summary statistics for each group (where appropriate) and (b) an effect estimate and its precision (e.g. confidence/credible interval), ideally using structured tables or plots.	Figure 2,3,4
Results of syntheses	20a	For each synthesis, briefly summarise the characteristics and risk of bias among contributing studies.	3.3 Risk of bias in individual studies, eFigure 1
	20b	Present results of all statistical syntheses conducted. If meta-analysis was done, present for each the summary estimate and its precision (e.g. confidence/credible interval) and measures of statistical heterogeneity. If comparing groups, describe the direction of the effect.	3.4 Analyses of overall effect
	20c	Present results of all investigations of possible causes of heterogeneity among study results.	3.4 Analyses of overall effect,
	20d	Present results of all sensitivity analyses conducted to assess the robustness of the synthesized results.	3.6 Sensitivity analyses
Reporting biases	21	Present assessments of risk of bias due to missing results (arising from reporting biases) for each synthesis assessed.	3.7 Risk of bias across studies
Certainty of evidence	22	Present assessments of certainty (or confidence) in the body of evidence for each outcome assessed.	3.4 Analyses of overall effect
**DISCUSSION**			
Discussion	23a	Provide a general interpretation of the results in the context of other evidence.	4.1 Summary of evidence, 4.2 Agreement with prior research
	23b	Discuss any limitations of the evidence included in the review.	4.3 External and internal validity, 4.4 Strengths and limitations

Section and Topic	Item #	Checklist item	Location where item is reported
	23c	Discuss any limitations of the review processes used.	4.3 External and internal validity,<br>4.4 Strengths and limitations
	23d	Discuss implications of the results for practice, policy, and future research.	4.5 Implications for further research<br>4.6 Implication for clinical practice
**OTHER INFORMATION**			
Registration and protocol	24a	Provide registration information for the review, including register name and registration number, or state that the review was not registered.	2. Methods
	24b	Indicate where the review protocol can be accessed, or state that a protocol was not prepared.	2. Methods
	24c	Describe and explain any amendments to information provided at registration or in the protocol.	n.a.
Support	25	Describe sources of financial or non-financial support for the review, and the role of the funders or sponsors in the review.	submission portal
Competing interests	26	Declare any competing interests of review authors.	submission portal
Availability of data, code and other materials	27	Report which of the following are publicly available and where they can be found: template data collection forms; data extracted from included studies; data used for all analyses; analytic code; any other materials used in the review.	online only supplements
